# Supplementary material for: A dietary sterol trade-off determines lifespan responses to dietary restriction in Drosophila melanogaster females
Source: eLife. 2021 Jan 26;10:e62335. doi: 10.7554/eLife.62335 (PMC7837700; doi:10.7554/eLife.62335)
Supplement: Supplementary file 1. — Decreasing doses of carbohydrate and increasing doses of protein resulted in significantly increased egg production. [file elife-62335-supp1.docx]

**Supplementary File 1.**

| **Variable** | **Estimate** | **Std. Error** | **t value** | **Pr (>Chisq)** |
| --- | --- | --- | --- | --- |
| Protein | 0.809 | 0.204 | 3.964 | < 0.001*** |
| Carbohydrate | -1.315 | 0.2178 | -6.035 | < 0.001*** |
