# Supplementary material for: A dietary sterol trade-off determines lifespan responses to dietary restriction in Drosophila melanogaster females
Source: eLife. 2021 Jan 26;10:e62335. doi: 10.7554/eLife.62335 (PMC7837700; doi:10.7554/eLife.62335)
Supplement: Supplementary file 2. — While variations in carbohydrate had no effect on lifespan, increasing doses of protein resulted in a significant change in lifespan. Visual inspection of the data agreed with our past experience with these diets (Piper et al., 2014; Piper, 2017) that the lifespan response was best modelled by the quadratic term for protein (Protein2) since lifespan peaked at our intermediate protein dose and fell away at both higher and lower doses. The quadratic term for Carbohydrate was thus also added to maintain balance in the model (Carbohydrate2). The interaction between protein and carbohydrate is not included in any of our analyses because these terms were not co-varied in a balanced way in our experimental design. [file elife-62335-supp2.docx]

**Supplementary File 2.**

| **Variable** | **Estimate** | **Std. Error** | **t value** | **Pr (>Chisq)** |
| --- | --- | --- | --- | --- |
| Protein | 7.088 | 0.642 | 11.6056 | < 0.001*** |
| Protein^2^ | -0.180 | 0.015 | -11.824 | < 0.001*** |
| Carbohydrate | -0.032 | 0.373 | -0.085 | 0.932 |
| Carbohydrate^2^ | 0.001 | 0.009 | 0.083 | 0.934 |
