# Supplementary material for: A dietary sterol trade-off determines lifespan responses to dietary restriction in Drosophila melanogaster females
Source: eLife. 2021 Jan 26;10:e62335. doi: 10.7554/eLife.62335 (PMC7837700; doi:10.7554/eLife.62335)
Supplement: Supplementary file 3. — Cholesterol had a significant positive effect on median lifespan, while diet type had a significant effect on median lifespan. Calories had no significant effect on lifespan. Data were analysed using a linear model with mixed effects, with vial as a random effect. [file elife-62335-supp3.docx]

**Supplementary File 3.**

| **Variable** | **Estimate** | **Std. Error** | **t value** | **Pr (>Chisq)** |
| --- | --- | --- | --- | --- |
| Calories | -0.001 | 0.017 | -0.395 | 0.693 |
| Cholesterol | 40.621 | 3.810 | 10.672 | < 0.001 *** |
| Diet type | -9.610 | 1.695 | -5.669 | < 0.001 *** |
