# Supplementary material for: A dietary sterol trade-off determines lifespan responses to dietary restriction in Drosophila melanogaster females
Source: eLife. 2021 Jan 26;10:e62335. doi: 10.7554/eLife.62335 (PMC7837700; doi:10.7554/eLife.62335)
Supplement: Supplementary file 4. — Cholesterol had a significant positive effect on cumulative eggs per female, while diet type had a significant effect on cumulative eggs per female. Calories had no significant effect on cumulative eggs per female. Data were analysed using a linear model with mixed effects, with vial as a random effect. [file elife-62335-supp4.docx]

**Supplementary File 4.**

| **Variable** | **Estimate** | **Std. Error** | **t value** | **Pr (>Chisq)** |
| --- | --- | --- | --- | --- |
| Calories | 0.002 | 0.022 | 7.751 | 0.350 |
| Cholesterol | 42.656 | 5.940 | 7.181 | < 0.001 *** |
| Diet type | -9.422 | 2.680 | -3.518 | < 0.001 *** |
