# Supplementary material for: A dietary sterol trade-off determines lifespan responses to dietary restriction in Drosophila melanogaster females
Source: eLife. 2021 Jan 26;10:e62335. doi: 10.7554/eLife.62335 (PMC7837700; doi:10.7554/eLife.62335)
Supplement: Supplementary file 5. — Data were analysed using a linear model with mixed effects, with vial as a random effect. [file elife-62335-supp5.docx]

**Supplementary File 5.**

| **Variable** | **Estimate** | **Std. Error** | **t value** | **Pr (>Chisq)** |
| --- | --- | --- | --- | --- |
| Protein | -2.21 | 5.882 | -3.764 | < 0.001*** |
| Protein^2^ | 3.732 | 1.448 | 2.578 | < 0.001*** |
| Carbohydrate | -2.365 | 2.592 | -0.912 | 0.362 |
| Carbohydrate^2^ | 1.794 | 6.248 | 2.871 | 0.072 |
| Cholesterol | 1.989 | 1.298 | 1.533 | < 0.001*** |
| Cholesterol^2^ | -1.607 | 1.122 | -14.324 | < 0.001*** |
| Protein: cholesterol | 1.801 | 1.560 | 11.545 | < 0.001*** |
| Protein^2^ : cholesterol | -4.135 | 3.800 | -10.882 | < 0.001*** |
| Carbohydrate^2^ : cholesterol | -2.763 | 5.154 | -5.362 | < 0.001*** |
