# Supplementary material for: A dietary sterol trade-off determines lifespan responses to dietary restriction in Drosophila melanogaster females
Source: eLife. 2021 Jan 26;10:e62335. doi: 10.7554/eLife.62335 (PMC7837700; doi:10.7554/eLife.62335)
Supplement: Supplementary file 6. — Data were analysed using a linear model with mixed effects, with vial as a random effect. [file elife-62335-supp6.docx]

**Supplementary File 6.**

| **Variable** | **Estimate** | **Std. Error** | **t value** | **Pr (>Chisq)** |
| --- | --- | --- | --- | --- |
| Protein | 5.598 | 0.752 | 7.443 | < 0.001*** |
| Protein^2^ | -0.137 | 0.018 | -7.523 | < 0.001*** |
| Carbohydrate | -0.253 | 0.129 | -1.956 | < 0.001*** |
| Cholesterol | 218.625 | 27.209 | 8.035 | < 0.001*** |
| Cholesterol^2^ | -343.733 | 35.242 | -9.754 | < 0.001*** |
| Protein : cholesterol | 6.239 | 2.189 | 2.850 | 0.004** |
| Protein^2^: cholesterol | -0.104 | 0.053 | -1.950 | 0.051 |
| Carbohydrate : cholesterol | -5.427 | 1.106 | -4.905 | < 0.001*** |
| Carbohydrate : cholesterol^2^ | 7.000 | 1.722 | 4.066 | < 0.001*** |
