# Supplementary material for: A dietary sterol trade-off determines lifespan responses to dietary restriction in Drosophila melanogaster females
Source: eLife. 2021 Jan 26;10:e62335. doi: 10.7554/eLife.62335 (PMC7837700; doi:10.7554/eLife.62335)
Supplement: Supplementary file 7. — Each of the main effects had a significant positive effect on egg production, and the amount of cholesterol significantly modified how P:C affected egg laying. Data were analysed using a linear model with mixed effects, with vial as a random effect. [file elife-62335-supp7.docx]

**Supplementary File 7.**

| **Variable** | **Estimate** | **Std. Error** | **t value** | **Pr (>Chisq)** |
| --- | --- | --- | --- | --- |
| P:C | 2.226 | 2.348 | 0.948 | < 0.001 *** |
| Cholesterol | 69.478 | 22.638 | 3.069 | < 0.001 *** |
| Cholesterol^2^ | -90.500 | 35.178 | -2.573 | < 0.001 *** |
| P:C : Cholesterol | 115.538 | 19.991 | 5.779 | < 0.001 *** |
| P:C : Cholesterol^2^ | -135.520 | 31.095 | -4.358 | < 0.001 *** |
