# Supplementary material for: A dietary sterol trade-off determines lifespan responses to dietary restriction in Drosophila melanogaster females
Source: eLife. 2021 Jan 26;10:e62335. doi: 10.7554/eLife.62335 (PMC7837700; doi:10.7554/eLife.62335)
Supplement: Supplementary file 8. — Cumulative egg production and cholesterol had a significant positive effect on median lifespan, while cholesterol2 had a significant negative effect on median lifespan. Data were analysed using a linear model with mixed effects, with vial as a random effect. [file elife-62335-supp8.docx]

**Supplementary File 8.**

| **Variable** | **Estimate** | **Std. Error** | **t value** | **Pr (>Chisq)** |
| --- | --- | --- | --- | --- |
| Cumulative eggs | 0.037 | 0.137 | 0.267 | 0.016 ** |
| Cholesterol | 115.620 | 27.262 | 4.241 | < 0.001 *** |
| Cholesterol^2^ | -148.892 | 45.292 | -3.287 | < 0.001 *** |
| Cumulative eggs : Cholesterol | 0.358 | 0.898 | 0.398 | 0.691 |
| Cumulative eggs : Cholesterol^2^ | 0.046 | 1.247 | -0.037 | 0.970 |
