# Supplementary material for: A dietary sterol trade-off determines lifespan responses to dietary restriction in Drosophila melanogaster females
Source: eLife. 2021 Jan 26;10:e62335. doi: 10.7554/eLife.62335 (PMC7837700; doi:10.7554/eLife.62335)
Supplement: Supplementary file 9. [file elife-62335-supp9.docx]

**Supplementary File 9.**

| Amino acids |  | FLYaa |
| --- | --- | --- |
| Essential amino acids | Phenylanine | 0.037 |
|  | Histidine | 0.026 |
|  | Isoleucine | 0.052 |
|  | Lysine | 0.057 |
|  | Leucine | 0.094 |
|  | Methionine | 0.025 |
|  | Arginine | 0.057 |
|  | Threonine | 0.056 |
|  | Valine | 0.062 |
|  | Tryptophan | 0.010 |
| Non essential amino acids | Alanine | 0.075 |
|  | Cysteine | 0.017 |
|  | Aspartate | 0.053 |
|  | Glutamate | 0.063 |
|  | Glycine | 0.062 |
|  | Asparagine | 0.047 |
|  | Proline | 0.052 |
|  | Glutamine | 0.046 |
|  | Serine | 0.079 |
|  | Tyrosine | 0.031 |
